# Endovascular Treatment of Acute Ischemic Stroke Due to Isolated Proximal Posterior Artery Occlusion

**DOI:** 10.3389/fsurg.2022.919509

**Published:** 2022-05-25

**Authors:** Guang Zhang, Yujing Zhu, Yeping Ling, Pingbo Chen, Jiaxing Dai, Chunlei Wang, Shancai Xu, Alina Shumadalova, Huaizhang Shi

**Affiliations:** ^1^ Department of Neurosurgery, The First Affiliated Hospital of Harbin Medical University, Harbin, China; ^2^ Department of General Chemistry, Bashkir State Medical University, Ufa, Republic of Bashkortostan, Russia

**Keywords:** acute ischemic stroke, endovascular treatment, neurological deficit, posterior cerebral artery, outcome

## Abstract

**Background:**

Acute ischemic stroke (AIS) due to isolated proximal posterior cerebral artery (PPCA) occlusion is rare but associated with high morbidity and mortality rates. However, the optimal treatment strategy for patients with AIS caused by PPCA remains unclear. We discuss our single-center experience with endovascular treatment (EVT) in patients with PPCA.

**Methods:**

Data from patients with AIS due to PPCA occlusion were retrospectively analyzed. We analyzed procedural details, the degree of reperfusion, functional outcomes, and complications. Functional outcomes were determined using the modified Rankin Scale (mRS) at 90 days, and good outcome was defined as mRS 0–2 at 90 days. Successful reperfusion was defined as modified treatment in cerebral ischemia (mTICI) 2b−3 after endovascular therapy. Safety variables included symptomatic hemorrhage (defined as an increase of four or more points in the National Institute of Health Stroke Scale score), vessel perforation or dissection, and new ischemic stroke in different territories.

**Results:**

Seven patients were included in this study. The mean age of the patients was 64 ± 12.4 years. Successful reperfusion was achieved in all seven patients (100%). Good outcomes were achieved at 90 days in 2 patients (28.6%), and favorable outcomes were observed in five patients (71.4%). One patient underwent angioplasty as rescue therapy after three attempts. One patient died because of severe gastrointestinal bleeding 24 h after EVT, which was probably a complication of intravenous alteplase. One patient had an embolism in the basilar artery and achieved complete reperfusion after rescue thrombectomy. Another patient had a complication of vessel dissection in the PPCA and underwent stent implantation as rescue therapy. We observed no recurrence of ischemic stroke or any intracranial hemorrhage on non-contrast computed tomography 24 h after the procedure.

**Conclusion:**

EVT may represent an alternative treatment strategy for patients with acute ischemic stroke caused by PPCA.

## Introduction

Endovascular treatment (EVT) is the standard of care for patients with acute ischemic stroke (AIS) caused by large vessel occlusion (LVO) in the anterior circulation (1). Previous studies have also indicated the benefits of EVT in basilar artery occlusion (2). Acute posterior cerebral artery occlusion accounts for 5%‒10% of all AIS cases (3). The territory of the proximal posterior cerebral artery (PPCA) usually has poor collaterals with a number of nerve fibers. PPCA occlusion involving thalamic perforating arteries, such as the Percheron artery, with abnormal variants can lead to severe neuropsychological deficits, visual symptoms, and unconsciousness (4, 5). However, no previous trials have focused on the effect of EVT on AIS due to PPCA occlusion (6, 7).

In this retrospective study, we aimed to present data on patients with AIS due to PPCA in clinical practice, focusing on the possibility of EVT as an alternative therapy in these patients.

## Methods

This study followed the Declaration of Helsinki and was approved by the Harbin Medical University ethics committee. Epidemiological, clinical and radiographical data were collected and reviewed. All patients or their legal guardians agreed to publication of clinical details and images. The participants provided their written informed consent to participate in this study.

**Figure 1:**
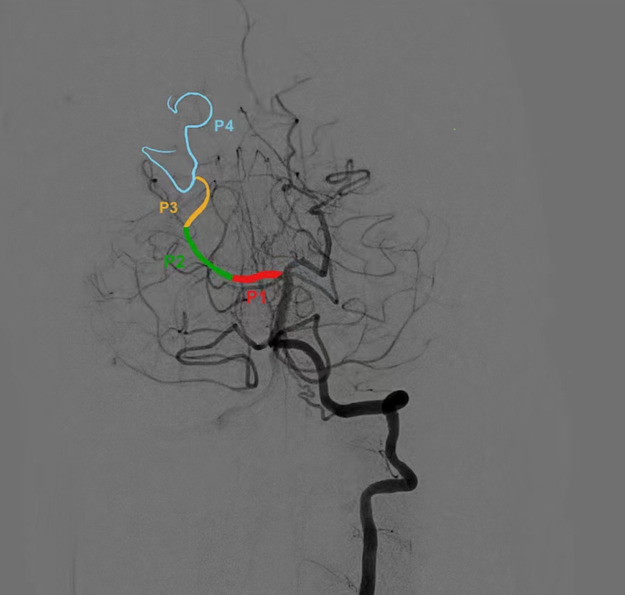
Segmentation of PCA Proximal posterior cerebral artery was defined as first and second segment of PCA. PCA, posterior cerebral artery.

All patients who underwent endovascular treatment for ischemic stroke at our institution were prospectively registered in an electronic database. For this study, we extracted data from patients with AIS due to PPCA occlusion between January 2020 and October 2021. The inclusion criteria were: (1) Acute occlusion of the PPCA, defined as the first and second segments of the posterior cerebral artery (8), as assessed by computed tomography (CT) angiography, magnetic resonance angiography (MRA), or digital subtraction angiography (DSA) before intervention; (2) Initiation of EVT within 24 h of symptom onset (Figure 1). The exclusion criteria were: (1) Severe preexisting disability, defined as a modified Rankin scale (mRS) score >2; (2) Secondary PPCA occlusion due to distal embolism during EVT of the basilar artery or vertebral artery occlusion; (3) Simultaneous occlusion of the anterior circulation, basilar artery, or intracranial vertebral artery.

The following data were extracted and analyzed: demographic characteristics, baseline National Institutes of Health Stroke Scale (NIHSS) scores, baseline Posterior Circulation Alberta Stroke Program Early CT Scores (pc-ASPECTS), history of previous stroke, use of intravenous thrombolysis, time from stroke onset to groin puncture, and procedure duration. We used the NIHSS score and Glasgow coma scale (GCS) to assess the severity of AIS at the time of symptom onset, and the NIHSS and GCS scores at 24 h and at 7 days post-EVT were also recorded. The baseline pc-ASPECTS score was evaluated by two experienced neurointerventionists using non-contrast CT (NCCT) or diffusion-weighted imaging (DWI) and followed by consensus adjudication. Procedural details, including the number of retriever attempts, rescue therapies, complications, such as perforation, and vessel dissection, were also collected.

### Technical Procedures

The procedure was performed under conscious sedation without heparinization. Femoral access was achieved, and a 6-French guide catheter was maneuvered into the dominant vertebral artery, and a cerebral angiogram was performed to confirm the PPCA occlusion. Under roadmap guidance, the microcatheter was navigated to the distal lumen of the occlusion site. A retrieval stent (Solitaire FR, ev3/Covidien, Irvine, CA, USA) of 4 × 20 mm was used in all cases. The stent retriever was left in place for 5 min to allow better clot integration and was retrieved through the guide catheter. Angiograms were obtained after each attempt. Repeated attempts, up to a maximum of three, were made until successful recanalization was achieved.

### Outcomes and Complications

The degree of reperfusion was assessed on the final DSA image using the modified treatment in cerebral ischemia (mTICI) score. Successful reperfusion was defined as an mTICI score of 2b−3 on the final angiogram. The main outcome was the mRS score at 90 days. A good outcome was defined as an mRS score of 0–2. A favorable outcome was defined as a score of 0–3. Improved neurological function was defined as a decrease in NIHSS score of ≥4. Complications included vessel perforation, arterial dissection, intracranial hemorrhage, stroke progression, and embolism in new territories. Intracranial hemorrhage was assessed using NCCT at 24 h after the procedure. Symptomatic hemorrhage was scored by the European Cooperative Acute Stroke Study TWO (ECASS II) and was defined as an increase in the NIHSS score of >3 points or a decline in the GCS score of >2.

### Statistical Analysis

Data were analyzed using descriptive statistics. Continuous variables are expressed as mean ± standard deviation or as medians and quartiles. Categorical variables are expressed as absolute values (number of patients) and relative frequencies (percentages).

## Results

### Patient Characteristics

A total of 343 patients with AIS underwent EVT between January 1, 2020, and October 31, 2021, and seven patients were diagnosed with isolated PPCA occlusion. The median patient age was 64 ± 12.4 years. All patients were male. Occlusions were located on the right side in four patients (57%). Three patients had intracranial artery atherosclerosis, one had cardio-embolism. Three patients received intravenous alteplase therapy preceded by EVT. Six patients (85.7%) were unconscious before the procedure, and the median baseline NIHSS score was 40 (range, 21–40). The median baseline GCS score was 7 (range: 7–10). The mean time from symptom onset to recanalization was 296.7 ± 160.2 min. The procedure duration ranged from 27 to 75 min. Patient characteristics are presented in Table 1.

**Table 1:** Clinical characteristics.

Patients number	1	2	3	4	5	6	7
Occlusion location	Left P1	Right P1	Left P1	Right P1	Right P1	Right P1	Left P2
Age	89	60	58	56	59	51	76
Hypertension	YES	NO	NO	NO	YES	YES	YES
Hypercholesterolemia	NO	NO	NO	NO	NO	NO	NO
Diabetes mellitus	NO	NO	NO	NO	NO	NO	NO
Previous stroke	NO	NO	NO	NO	YES	NO	YES
Atrial fibrillation	NO	NO	NO	NO	NO	NO	YES
Smoking	Yes	NO	NO	NO	Yes	NO	NO
Baseline NIHSS	40	38	36	40	40	40	21
Baseline ASPECTS	10	9	7	10	9	9	8
pre mRS	0	0	0	0	0	0	0
Baseline GCS	7	7	8	7	9	7	10
Intravenous thrombolysis	YES	NO	YES	NO	NO	NO	YES
Time from onset to groin puncture (min)	285	128	149	108	540	123	390
Time from puncture to recanalization (min)	40	62	75	30	50	70	27

*P1, first segment of the posterior cerebral artery; P2, second segment of the posterior cerebral artery; NIHSS, National Institutes of Health Stroke Scale; ASPECTS, Alberta Stroke Program Early CT Score; mRS, modified Rankin scale; GCS, Glasgow coma scale.*

### Outcomes and Complications

Patient outcome and procedure comlications are presented in Table 2. Successful reperfusion was achieved in all seven patients (100%). Two patients (28.6%) achieved complete reperfusion (mTICI: 3). One patient underwent angioplasty as rescue therapy after three failed retrieval attempts. Good outcomes were achieved in two patients (28.6%) and favorable outcomes were observed in five patients (71.4%). The mortality rate was 14.3%. Five (71.4%) patients had improved neurological function at 24 h post-procedure. One patient died because of severe gastrointestinal bleeding 24 h after EVT, probably as a complication of intravenous alteplase. The incidence of periprocedural complications was 28.6%. One patient had an embolism in the basilar artery and achieved complete reperfusion after rescue thrombectomy. Another patient had a complication of vessel dissection in the PPCA and underwent stent implantation as rescue therapy. Endovascular procedure-related complications were not observed in the remaining five patients. We observed no recurrence of ischemic stroke or any intracranial hemorrhage on follow-up NCCT 24 h after the procedure.

**Table 2:** Outcomes and complications.

Patients number	1	2	3	4	5	6	7
mTICI score	2b	2b	2b	3	3	2b	2b
Infarct location on follow up images	NA	Thalamus	Midbrainand thalamus	Occipital lobe	Occipital lobe	Thalamus and occipital lobe	Thalamus
Asymptomatic hemorrhage	NA	NO	NO	NO	NO	NO	NO
NIHSS at 24 h	NA	36	25	0	8	15	10
GCS at 24 h	NA	8	9	15	15	12	13
mRS score at 90 days	6	3	3	0	1	4	3

*mTICI, modified Treatment in Cerebral Ischemia; National Institutes of Health Stroke Scale; mRS, modified Rankin Scale; GCS, Glasgow coma scale.*

### Case Illustration

#### Case 1

**Figure 2:**
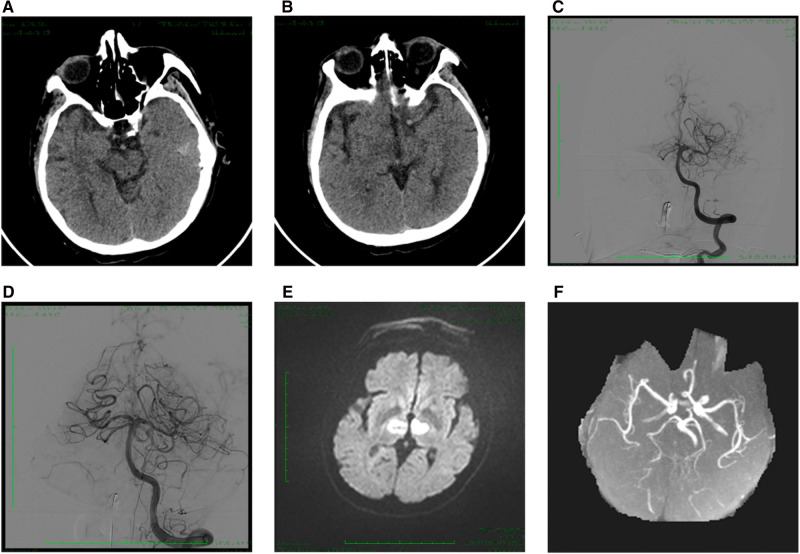
Case 1. (**A,B**) Baseline CT indicated pc-ASPECTS score was 9. (**C**) Anterior-posterior angiogram revealed an occlusion of right PPCA. (**D**) Post-thrombectomy angiogram indicated the mTICI score was 2b. (**E**) Post-procedural DWI showed acute bilateral thalamic infarction. (**F**) MRA showed successful recanalization of right PPCA at 48 h after procedural. PPCA, proximal posterior cerebral artery; pc-ASPECTS, posterior circulation Alberta Stroke Program Early CT Score; mRS, modified Rankin Scale.

Patient (No. 2) was admitted to the hospital with left limbs hemiplegia and dysarthria for 4.5 h and received intravenous alteplase (0.9 mg/Kg). Twelve h after admission, his consciousness deteriorated. His NIHSS and GCS scores were 38 and 7, respectively. NCCT did not reveal any intracranial hemorrhage. The baseline pc-ASPECTS score was nine (Figures 2A,B). DSA demonstrated occlusion of the right PPCA (Figure 2C), and the pre-mTICI score was 0. Endovascular procedures were performed with a 4 × 20-mm retrieval stent (Solitaire FR, ev3/Covidien). The final mTICI score was 2b after two attempts (Figure 2D). The time from onset (disturbance of consciousness) to groin puncture was 128 min, while the duration of the procedure was 62 min. Tirofiban was administered for 24 h, followed by aspirin (100 mg/day) and clopidogrel (75 mg/day). Post-procedural DWI indicated infarction in the bilateral thalamus (Figure 2E). MRA indicated complete recanalization of the right PCA (Figure 2F). The mRS score on day 90 was 3.

#### Case 2

**Figure 3:**
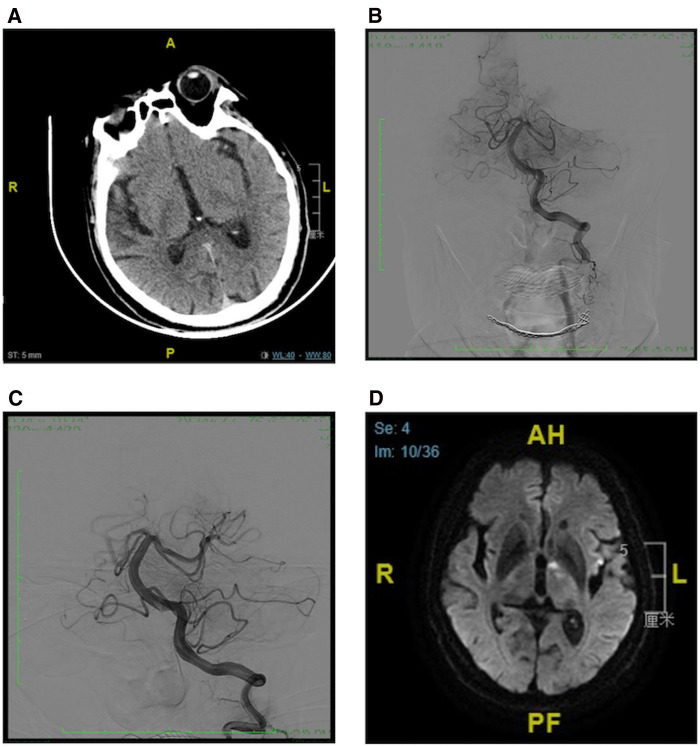
Case 2. (**A**) Baseline CT indicated pc-ASPECTS score was 10, (**B**) Anterior-posterior angiogram revealed an occlusion of left PPCA. (**C**) Post-thrombectomy angiogram indicated the mTICI score was 2b. (**D**) Post-procedural DWI showed acute left thalamic infarction. PPCA, proximal posterior cerebral artery; pc-ASPECTS, posterior circulation Alberta Stroke Program Early CT Score; mRS, modified Rankin Scale.

Patient (No. 7) was admitted to the hospital with right hemiplegia and left oculomotor paralysis for 5 h. His NIHSS and GCS scores were 21 and 8, respectively. NCCT did not reveal any intracranial hemorrhage. The baseline pc-ASPECTS score was ten (Figure 3A). DSA demonstrated occlusion of the left P2 segment (Figure 3B), and the pre-mTICI score was 0. Endovascular procedures were performed with a 4 × 20-mm retrieval stent (Solitaire FR, ev3/Covidien). The final mTICI score was 2b after one attempt (Figure 3C). The time from symptom onset to groin puncture was 390 min, while the duration of the procedure was 27 min. Post-procedural DWI indicated infarction in the left thalamus (Figure 3D). The mRS score on day 90 was 3.

## Discussion

Since publication of five randomized clinical trials, EVT has become the standard therapy for patients with AIS caused by large-vessel occlusion in the anterior circulation artery (9–13). In this retrospective study, we found that EVT may be a suitable alternative treatment for PPCA occlusions. The proportion of successful reperfusions in our cases was 100%, and 28.6% of the patients achieved good functional outcomes at 90 days, with a low rate of procedure-related complications.

The perforating arteries from the P1 segment and junction of the P1‒P2 supply blood to the upper midbrain, thalamus, and hypothalamus. The territory of the P2 segment includes the lateral geniculate corpus and the visual radiation adjacent to the temporal lobe (4). Therefore, occlusion of the PPCA often leads to infarction of the midbrain, thalamus, temporal lobe, and occipital lobe. Patients often present with contralateral hemiplegia and visual field defects, and have a poor outcome. Therefore, it seems necessary to provide more aggressive treatments for patients with AIS due to PPCA occlusion. In this study, six of seven patients lost consciousness after symptom onset, demonstrating that PPCA occlusion could lead to severe clinical manifestations.

In line with previous studies, 28.6% and 71.4% of patients achieved good or favorable outcomes at 90 days in this study, respectively, indicating that PPCA is an alternative therapy with an acceptable outcome. Meier et al. reported nine patients with PCA occlusion who underwent intra-arterial thrombolysis, and 67% of the patients had a favorable outcome (14). Strambo et al. compared the cognitive, visual, and disability outcomes of EVT, intravenous thrombolysis, and the best medicine therapy for isolated PPCA occlusion. Their study revealed a higher ratio of recanalization and favorable outcomes in the EVT group, and EVT did not significantly increase mortality (15). Previous studies have revealed a high successful recanalization rate, between 80% and 100%, with a low procedural complication rate of approximately 4%–7%. Good outcomes were observed in 59‒66% of all patients at 90 days after EVT (16–18). Memon et al. (16) and Herweh et al. (19) presented the results of studies of 15 and 23 patients with isolated PPCA occlusion, respectively, who were treated with EVT. The successful recanalization rates were 80% and 54%, respectively. The proportions of patients with good outcomes at 90 days were 60% and 43.5%, respectively. Memon et al. reported that three patients (20%) experienced hemorrhage after the procedure, and one (6%), who had symptomatic hemorrhage, died. Herweh et al. reported a complication rate of 26% and a mortality rate of 13% within 90 days. In our study, we found one patient with asymptomatic subarachnoid hemorrhage, one patient with an embolism in a new territory, and one patient with vessel dissection, indicating the safety of EVT involving the PPCA.

In our study, we summarized the data of patients with PPCA occlusion who had undergone EVT in a single center. After evaluating the feasibility of EVT, we observed that all patients achieved successful reperfusion. Although one patient had an embolism in the basilar artery, the patient finally achieved complete reperfusion. Patient 1 received bridge therapy and died of severe upper gastrointestinal bleeding at 24 h after EVT. The patient received 0.9 mg/kg rt-PA for intravenous thrombolysis, followed by EVT. Systemic heparinization was not performed during the procedure, and as the reason for stroke was not large artery atherosclerosis, tirofiban or other antiplatelet agents were not administered during and after the procedure. Therefore, we speculated that the reasons for severe upper gastrointestinal bleeding included older age, poor general condition, and coagulopathy caused by the use of rt-PA. In the remaining six patients, no operation-related complications were observed during hospitalization or follow-up. Although our study had a high rate of successful recanalization, our good clinical outcomes were worse than those of other studies. This was because most of our patients were in coma and their baseline NIHSS scores were much higher than those of patients in the previous studies, although the neurological deficits improved in most patients after EVT.

The present study has some limitations, including its small sample size. In addition, although we extracted data from a prospective database, the case series was retrospective in nature. Third, we only analyzed patients with ischemic stroke involving PPCA occlusion who accepted EVT; i.e., those who accepted only medicine were not included in our study. Forth, patients with PPCA occlusion but who had mild symptoms might not undergo angiographic evaluation and may thus not have undergone EVT. Finally, we missed the details of these thrombus, such as thrombus length, pathologic information which potentially help to understand the etiology of PPCA occlusion.

In conclusion, our study suggests that EVT may represent an alternative treatment strategy for patients with AIS caused by PPCA occlusion. Nevertheless, further randomized studies are urgently required to verify our findings.

## Data Availability

The original contributions presented in the study are included in the article/Supplementary Material, further inquiries can be directed to the corresponding author/s.
